# The General Growth Tendency: A tool to improve publication trend reporting by removing record inflation bias and enabling quantitative trend analysis

**DOI:** 10.1371/journal.pone.0268433

**Published:** 2022-05-20

**Authors:** Joost L. D. Nelis, Gonçalo Rosas da Silva, Jordi Ortuño, Aristeidis S. Tsagkaris, Benny Borremans, Jana Haslova, Michelle L. Colgrave, Christopher T. Elliott

**Affiliations:** 1 CSIRO Agriculture and Food, St Lucia, Queensland, Australia; 2 Institute for Global Food Security, School of Biological Sciences, Queen’s University, Belfast, United Kingdom; 3 Department of Food Analysis and Nutrition, Faculty of Food and Biochemical Technology, University of Chemistry and Technology Prague, Prague, Czech Republic; 4 Department of Ecology and Evolutionary Biology, University of California Los Angeles, Los Angeles, California, United States of America; Universitat de Barcelona, SPAIN

## Abstract

The trend of the number of publications on a research field is often used to quantify research interest and effort, but this measure is biased by general publication record inflation. This study introduces a novel metric as an unbiased and quantitative tool for trend analysis and bibliometrics. The metric was used to reanalyze reported publication trends and perform in-depth trend analyses on patent groups and a broad range of field in the life-sciences. The analyses confirmed that inflation bias frequently results in the incorrect identification of field-specific increased growth. It was shown that the metric enables a more detailed, quantitative and robust trend analysis of peer reviewed publications and patents. Some examples of the metric’s uses are quantifying inflation-corrected growth in research regarding microplastics (51% ± 10%) between 2012 and 2018 and detecting inflation-corrected growth increase for transcriptomics and metabolomics compared to genomics and proteomics (Tukey post hoc p<0.0001). The developed trend-analysis tool removes inflation bias from bibliometric trend analyses. The metric improves evidence-driven decision-making regarding research effort investment and funding allocation.

## Introduction

**Fig 1:**
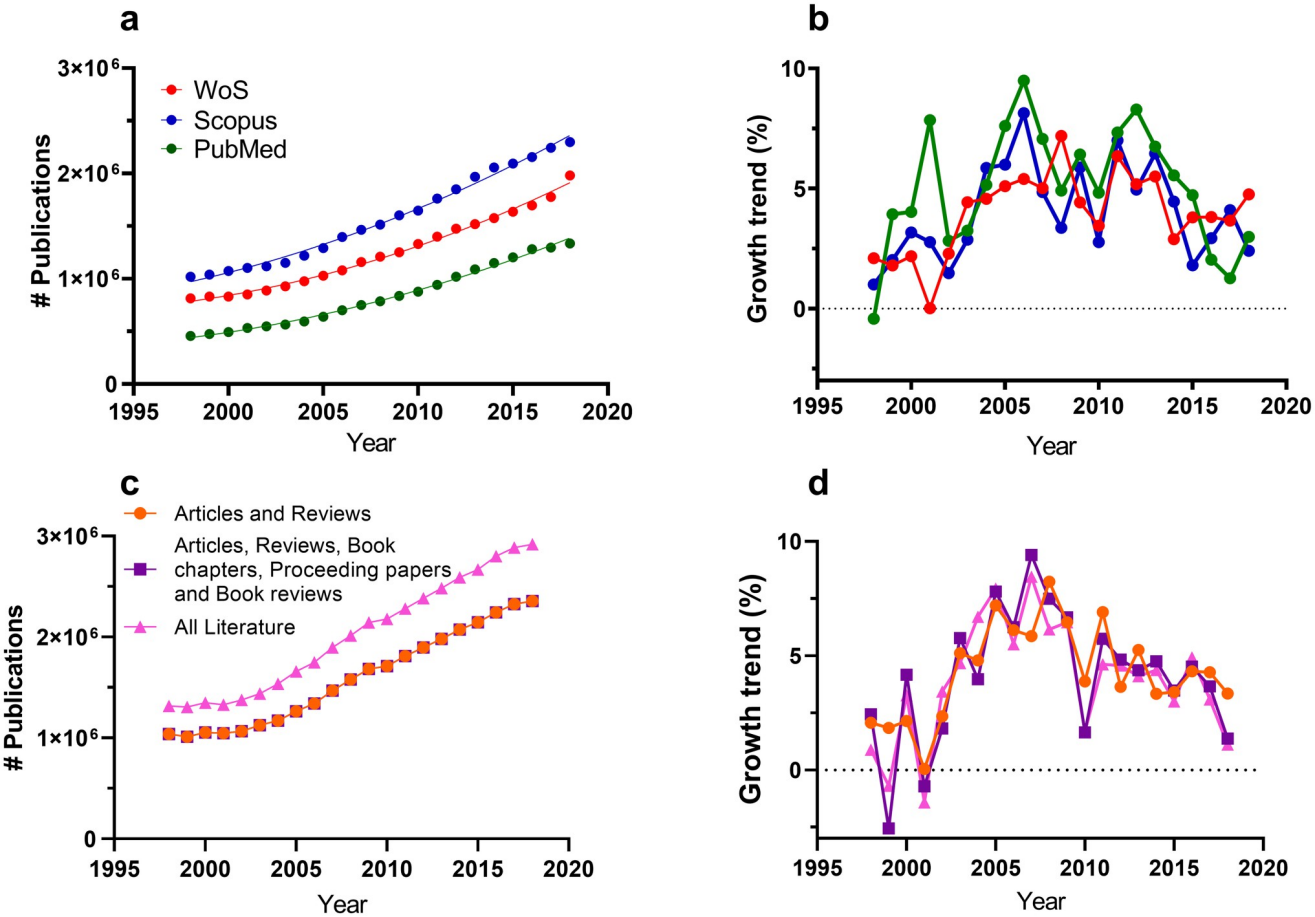
Trend of the overall publication output in science. Total publications (a) and yearly percentage increase (b) on WoS excluding ESCI index (red), Scopus (blue) and PubMed (green) databases for all scientific publications published between 1998 and 2018. Lines in (a) are fitted quadratic trend lines. Lines in (b) connect individual data points. Search term used in WoS was: “PY = 1998–2018”. In Scopus: PUBYEAR AFT 1998. In PubMed 1998:2018[dp]. Searches were refined for articles and reviews only. (c) shows total publication output in WoS excluding ESCI index (search term “PY = 1998–2018”) refined for articles and reviews (orange); articles, reviews, book chapters, proceedings papers and book reviews (purple) or all scientific literature listed in WoS (pink). (d) shows yearly percentage increase for the total publication output using the data shown in (c).

The chronological distribution of publications on a given field, commonly called “publication trend”, is often included in critical, systematic and bibliometric reviews as well as in grant applications. This metric is used to illustrate the rise in scientific interest in the discussed field, identify key knowledge gaps within a larger field and highlight the commercial potential of technologies [1–6]. Such trend analyses can have important implications in the decisions made by researchers and funders to invest in a specific field [7]. It is thus paramount that reported trends accurately reflect true field-specific growth. However, the use of the unadjusted publication trend (i.e., number of articles over time) has several drawbacks. Firstly, the general growth in the total number of publications across all disciplines and fields may influence field-specific growth trends. Scientific publication output (reviews and original research articles) in the leading scientific databases has grown significantly over the past two decades. This is reflected by the quadratic trends observed for Scopus (R^2^ = 0.993), Web of Science (WoS) (R^2^ = 0.994) and PubMed (R^2^ = 0.995) (Fig 1a). This increase in total publications across science affects the growth rates for specific fields. Secondly, the current method lacks quantitative information, making it hard to compare trends between fields. For instance, research on estuary toxicological pollution [8] and microplastic pollution in the marine environment [9] are two fields that significantly increased during the last two decades. However, it is very difficult to determine which of the two is growing more rapidly by simply comparing the publication trends presented [8, 9].

Different approaches can be adopted to resolve these shortcomings. As shown in Fig 1b, the average yearly percentage increase in publications in WoS (3.99% ±0.37% SEM), Scopus (4.16% ± 0.43%) and PubMed (5.26% ± 0.55%) was similar between 1998 and 2018. These figures corroborate findings (using WoS, Scopus, Microsoft academics and Dimensions databases) indicating an average overall growth in the scientific literature of 5.08% since 1952 until present day [10]. These overall growth rates may be used to correct field-specific growth rates for total publication inflation, similar to the widely used approach in macroeconomy to adjust the gross domestic product (GDP) for inflation [11]. Such an approach can remove the bias derived from the yearly total publication increase from the specific field growth and enable quantitative comparison between different fields.

This paper discusses the potential of this approach as well as other approaches to resolve the discussed shortcomings of publication trend analyses. After comparing approaches across randomly chosen life science topics (37) and published reviews (26), we proceed to introduce a novel metric. This metric, the General Growth Tendency (or GGT), calculates inflation-corrected, field-specific yearly growth rates. The benefits of the GGT are demonstrated by applying the metric to datasets of previously published reviews and other well-known life sciences fields. Finally, we assess additional applications such as comparing trends within research fields that are corrected for field-specific inflation and obtaining inflation-corrected patent publication trends.

## Methods

### GGT calculation and interpretation

The GGT for a specific research field or technology was calculated by subtracting the annual percentage growth rate in the overall literature from the annual increase/decrease in the research field of interest, as shown in Eq (i):

$$GGT=\left(\left(\frac{PS_{y}-PS_{y-1}}{PS_{y-1}}\right)-\left(\frac{PO_{y}-PO_{y-1}}{PO_{y-1}}\right)\right)*100\%$$ (i)

Where:

PS–Number of publications in the research field of interest

PO–Overall number of publications (articles and reviews)

y–Year

Succinctly, a GGT of 5% for a given year signifies that the number of articles for that research field grew 5% faster than the overall body of scientific literature, relative to the previous year.

It is unlikely that well-established fields exhibit more than a ~2 fold increase relative to the total publication growth (which fluctuates around ~4% in WoS; Fig 1b). Many GGTs will therefore fall within ± 10%. If a field has a GGT>100% the yearly increase or decrease in publication output in that field is roughly 20 times higher than what could be expected from growth caused by publication inflation. This may occur for fields with small absolute numbers of yearly publication outputs leading to high GGT fluctuations. However, such large fluctuations are unlikely to occur in established fields with higher absolute numbers of yearly publication output. Thus GGT>100% or fluctuations in the GGT of more than 100% likely represent unsustainable growth or large fluctuations due to low numbers of publication outputs. Rolling averages were considered to compensate for erratic patterns but were not implemented due to the difficulty to perform statistical analyses on such data and the risk of creating misleading patterns in the data. Rolling averages can create the impression of trends by muting year-to-year variability and amplifying any large random shift to a high or low value. As such, correlation calculations (be they linear or nonlinear regression), on moving average data would be unsuitable, because the used values are not completely independent.

It should be noted that restrictions on publication type (reviews and articles only) were chosen to avoid article duplication from pre-prints and conference papers. The document types used for PO calculation in Eq (i) was kept constant. The absolute total amount of publications remains almost identical when articles, reviews, book chapters, proceeding papers and book reviews are included instead of articles and reviews for PO (Fig 1c; S2 File). The absolute number of publications changes if all literature in WoS (meeting abstracts, editorial material, corrections, letters etc.) is included. Interestingly, changes in the material inclusion for PO do not significantly affect the growth trend (One-Way ANOVA; P = 0.89) which remains fluctuating around 4% even if all literature is included (Fig 1d). As such, changing the document type choice was considered to have an insignificant effect on the baseline used for correcting publications inflation. Nonetheless, the PO can be changed (for instance including conference papers) to reflect field specific output format preferences (conference papers are the preferred output format in some fields) for more detailed field specific inflation correction if desired.

### Data collection

Twenty-six review articles that included a publication output trend analysis were randomly selected from reviews covering life science fields found in WoS with the only criteria being that the review needed to have a publication output trend analyses on a field in the life sciences, and a specified keyword search allowing replication. These open selection criteria were applied to avoid any bias in the selection of such reviews. The publication output trends reported in these articles were reconstructed by utilizing each review’s choice of search strings, article types and database. The yearly publication outputs obtained (S1 File) were used to calculate GGTs and inflation adjusted trend lines. Additionally, GGTs, adjusted and unadjusted trend lines were calculated for another 37 life-science related fields including diverse fields such as medicine, biotechnology, food technology and ecology (Table 1; S2 File). Here, fields were specifically selected to include both relatively young and fast growing (e.g machine learning and microplastics) and more established fields (e.g. Alzheimer’s Disease and vaccines). Less common fields (such as food authentication, edible insects and cell-based meat) were also included to cover a wide range of possible datasets. Search strings were limited to peer-reviewed original articles and reviews only using the WoS database, unless if the field commonly uses other forms of output (e.g., in the field of computer science proceedings papers are often used). Such cases are mentioned specifically.

**Table 1:** Investigated fields in the life sciences. The 37 fields for which GGTs were calculated and inflation adjusted trend analysis performed. Fields are classified into different sections. The classification used is non-stringent and merely used to reflect the potential of GGT over a variety of disciplines in the life-sciences.

Medicine/Health	Biotechnology	Food technology/safety	Ecology
Alzheimer	Machine learning	Food authenticity	Phytochemistry
Microbiome	Metabolomics	Food fraud	Disease ecology
Obesity	Transcriptomics	Pesticides	Macroecology
NAFLD	Proteomics	Food packaging	Invasion biology
Mitochondria	Genomics	Cannabinoids	Population ecology
Autophagy		Edible insects	Cross-species transmission
Immunotherapy		Seaweed (food/feed)	Point-of-care
Metal health		Cell-based meat	
Vaccines		Prions	
Fetal and Neonatal		Dioxins	
Coronavirus		Avian flue	
Microplastics		Melamine	
Antibiotic resistance			

WoS was chosen over PubMed due to its more frequent use for bibliometric work, wider range of disciplines, and more careful curation compared to Scopus and Google Scholar, which have more issues with redundancy [12, 13]. In all cases, data collection was undertaken within 1 week to minimize fluctuations in the database. GGTs were also calculated for two patent groups within the life sciences. A spreadsheet with the granted patents per field of technology and per country of residence for each year from 2010 until 2019 was downloaded from the European Patent Office (EPO) (www.epo.org; last accessed 23/10/2020) and used to calculate patent GGTs. The raw data and yearly GGTs of all selected reviews and fields discussed here as well as the search strings are specified in the S1 and S2 Files.

**Fig 2:**
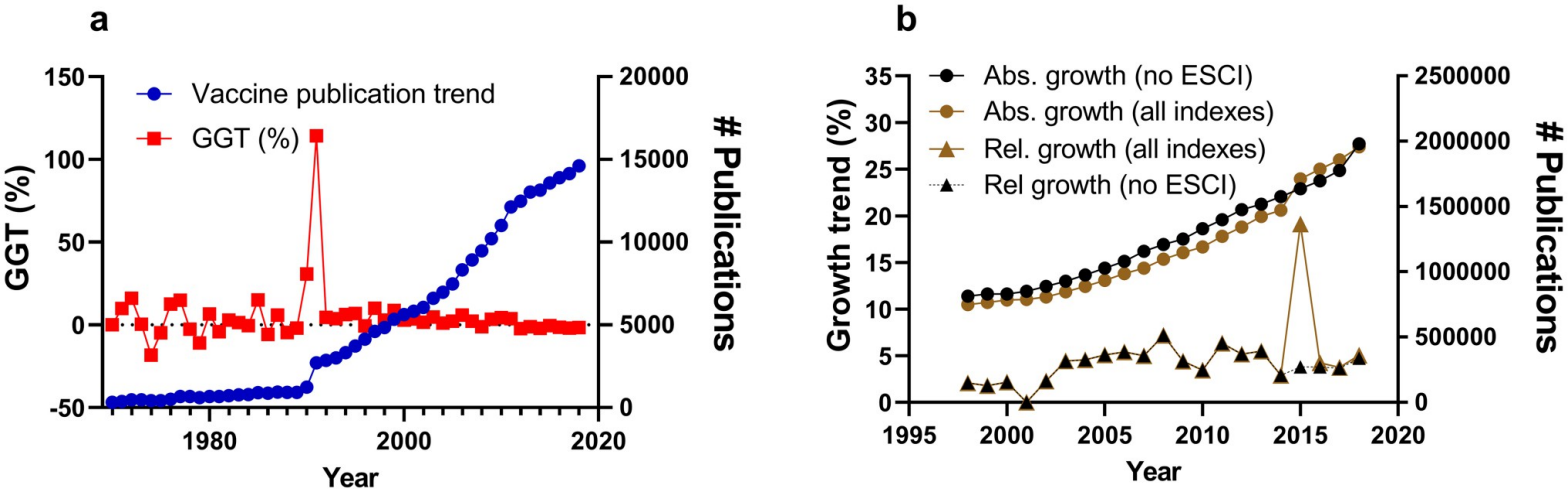
The effects of index changes. a) The publication trend (blue circles) for articles and reviews (right-axes) and GGT (red squares; left-axes) for the search string: TS = (vaccin*) AND PY = (1970–2020) on WoS excluding the ESCI index. b) The absolute number of publications (right y-axes; circles) and relative yearly percentage increase (left y-axes; triangles) in total publications reported on WoS using all indexes (brown) of WoS or all but the ESCI index (black). Spikes in the GGTs and growth trends indicate the inclusion of keyword searches in abstracts in 1991 (a) and the inclusion of the ESCI index in 2015 (b) respectively.

The final period of analysis typically covered 1996–2018, although it was adapted in some cases in relation to the field’s age. The years 2019–2021 were excluded as these records will continue to be updated. GGT calculation was restricted to publications after 1991 since the WoS search algorithm includes keyword searches in abstracts from 1991 onwards only, which creates an artefact in field-specific analyses before and after 1991 making direct comparison before and after 1991 unfeasible [14] (Fig 2a). Similarly, the inclusion of the Emerging Sources Citation Index (ESCI) index in WoS from 2015 [15] implied the detection of another artefact observed in the total publication output, when calculating the GGT for this year (Fig 2b). For that reason, the use of ESCI was avoided for the GGT calculation for fields. However, the ESCI index was included (and the year 2015 excluded) for the search strings that were identical to the analysed reviews to keep maximum similarity with the data presented in those reviews. It should be noted that these results highlight how shifting indexing policies can distort the results of bibliometric analyses and how the GGT can aide to identify these issues. An Excel calculator (S3 File) and an R script (S4 File) with instructions are provided alongside this paper to facilitate the GGT calculation for large quantities of data.

### Statistical analysis

GGTs of two or various fields were compared over a fixed period using the yearly GGT values as replicas using T-tests or one-way ANOVA and Tukey’s post-hoc comparisons. For trend analyses, Spearman’s correlation was used to determine if there was a monotonic component to the association and if the correlation was negative or positive. Spearman’s correlation was chosen instead of Pearson’s correlation since the former does not require the assumption that the data is neither monotonic nor are the sets large enough to be affected by the Central Limit Theorem. Descriptive statistics were used to indicate the basic features of the data. Descriptive statistics were calculated using Excel and inferential statistics using Graphpad V8.0. Standard Error of the Mean (SEM) is used to reflect sample distribution.

## Results and discussion

### Critical comparison of the GGT with other metrics

A number of studies have highlighted the bias in crude publication trends, and alternatives to the GGT can be found in the literature. Giraldo et al. suggested the calculation of ratios for field-specific (R_1_) and total publication (R_2_) output for two timeframes with R_1_ = (field 1 for timeframe A/ field 1 timeframe B) and R_2_ = (total publication output for time frame A/ total publication output for frame B). In this way, one can compare R_1_ and R_2_ to detect higher or lower publication output increases for the field compared to the total general output [16]. However, this method requires the use of several years of data to obtain average R values ranging over timeframe A and B. This limits the number of data points present in the trend analyses of the R values. Narotsky et al. [17] compared the ratio of average, field-specific output/total publication output between different periods, which similarly removes data points from the trend analyses and may result in misinterpreting changes due to random variation. Additionally, it is difficult to compare trends between fields with different absolute publication outputs with this system since the ratios will vary significantly. In some of the review papers analysed, the authors used an exponential function as a proxy for the publication trend for the specific field of study [18]. Therefore, we considered calculating the ratio R_trend_ of the field-specific publication trend slope and the slope of the overall total publication output obtained after fitting the yearly publication output data. According to this metric, R_trend_<1 would indicate a decrease, R_trend_ = 1 no increase and R_trend_>1 an increase in field-specific publication output relative to the total publication output. However, this metric would not be able to detect brief periods of intensified or reduced research activity. Moreover, the growth trends may follow different models (linear, quadratic or exponential), making it difficult to compare with the underlying overall publication trend. Finally, normalization of the publication output on a 1–100% axis was considered. This may enable visual comparison of the trends between various fields and the general publication output as well. However, in this scenario the trends themselves are not quantified, and the relationship between the field-specific and general publication trends may be difficult to analyse. Therefore, the GGT can be considered to be more informative than other described metrics as it offers not only an unbiased value that corrects for total publication growth but also avoids over-generalization of the growth trends and enables quantitative statistical analyses within and between field-specific trend lines. Additionally, inflation correction procedures are also proposed for citation analyses [19]. In theory, the principle behind the GGT may be used for citation analyses as well whereby the steady increase in yearly citation output in a specific field can be corrected for the overall increase in citation output throughout the scientific literature. However, the usefulness of such a measure would need to be studied in more detail as the focus here was on publication output. Such approaches can equally be beneficial to compare shifts in citation practices over time. Segmented regression analysis is equally suggested as a statistical tool to analyse crude publication trends [20]. Such regression analyses may aide to tease apart differences in publication trends or the relative growth of these trends over time and is thus complementary to what is proposed here.

### Comparing the GGT with unadjusted publication trends using data from published reviews

GGTs for the fields discussed in the 26 reviews were calculated. In some cases, the reported keyword search did not match the obtained number of publications and minor changes (mainly adding Boolean operators) to the search string were required, complicating replication. However, keyword searches identical to those in various reviews could be used directly with minimal differences and the publication and GGT trends for a selection of these studies [1, 5, 6, 17, 21–23] which are shown in Fig 4a–4f enabling direct comparison between the yearly publication output analyses reported in the reviews and the GGT analyses. Data and search strings are given in the S1 File. All datasets showed an overall increase in the yearly number of publications and several studies concluded that the field increased considerably [1, 5, 6, 22, 23]. For instance, articles on the study of malaria [23] and giardia [22] grew considerably during the analyzed period (Fig 4b, 4c). Nonetheless, the GGTs calculated for those datasets fluctuated around zero (GGT = -0.8% ± 2.3% for [22] and 0.9% ± 2.3% for [23]). Likewise, a study published in Trends in Analytical Chemistry reported an increasing trend in publications in the sensor arrays field during 2008–2018 (Fig 4d) [1]. However, the average GGT calculated over these years was 2.3% ± 1.7%, suggesting little growth relative to total publication growth. Correlation coefficients can also be calculated to test whether the GGT is consistently significantly different from zero, as this would mean that a field is continually growing/shrinking faster than the reference field. For example, the correlation coeficient calculated for the sensor array GGTs over 1998–2018 suggest (although not significantly) that there may even have been a decrease in inflation corrected growth (r = -0.63; p = 0.12). The examples described above are a practical demonstration of the bias due to the effect of total publication growth on a specific field trend and the benefits of GGT to correct it.

**Fig 3:**
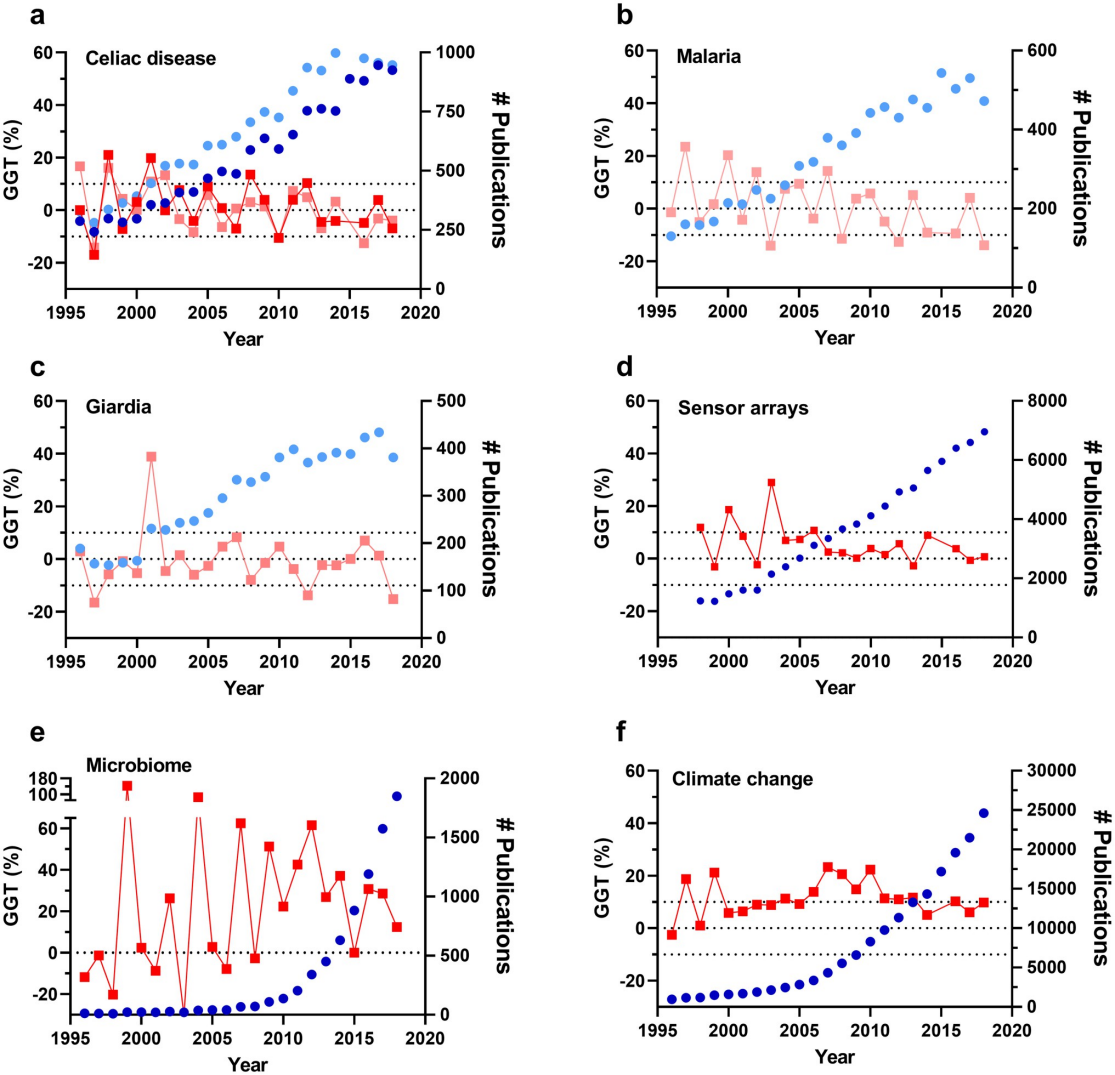
Trend analyses of publication trends derived from reviews. Fields analysed are: (a) celiac disease [17, 21], (b) Malaria [23], (c) Giardia [22], (d) sensor arrays [1], (e) microbiome [6] and (f) climate change [5]. All searches were conducted using keyword searches in the field section of WoS (dark colours) or PubMed (light colours). Search terms used were identical to those specified in the articles (see S1 File for specifications). The GGTs (dark and light red squares) are plotted on the left y-axes. The number of publications per year (dark and light circles) are plotted on the right y-axes.

Reviews covering celiac disease (Fig 3a) also reported an increase in the unadjusted publication trend, albeit more nuanced [17, 21]. Narotsky et al. [17] reported an increase in the field-specific/total output ratio for 1995–1999 compared to 2000–2004 and 2005–2009 concluding that publications covering celiac disease increased ~0.2% relative to the overall scientific production over that period. Nevertheless, the GGT trend (7.5% ± 5.1%; 2.5% ± 3.7%; -0.7% ± 2.5% for the respective periods) indicates that this increase is likely due to random variation around zero inflation-corrected growth. Indeed, a one-way ANOVA comparing GGTs of these periods showed no significant difference (p = 0.63) in inflation-corrected growth during the different periods. Moreover, a significant negative correlation was found over the 1995–2018 period (r = -0.43; p = 0.04). Another review on the same field reported that the publications doubled between 1996 and 2018, which contributed to improvements in diagnostic tests and histopathology [21]. However, the total number of publications in WoS during the same period increased ~2.6 fold. Again, the calculated GGT (1996–2018) showed a fluctuation around zero (GGT = 1.5% ± 2.2%) and no significant correlation or deviation from zero (r = -0.11; p = 0.63), thus reflecting that the bias of total growth can lead to inaccurate conclusions. The GGTs and yearly number of publications also largely overlap between the studies even though different databases were used. Therefore, the observed increases in yearly publications for studies covering celiac disease may be solely attributed to the overall increase in publication growth.

In other cases, the growth observed in the unadjusted publication trend does reflect field-specific growth. For instance, the exponential growth observed for microbiome research in the review by Jones [6] was corroborated by the calculated GGT over 2008–2018 (31% ± 6%) (Fig 3e). This GGT can be interpreted as an increased annual growth of ~30% in this field relative to the overall scientific production during that period. In the same line, the field showing the highest number of publications was climate change with stable GGTs of 12% ± 2% and no significant changes in the GGT over the period 2008–2018 (r = 0.15; p = 0.52) reflecting the field is continually growing faster than the reference field (Fig 3f). These findings indicate that the scientific community invested considerable efforts in both fields [5, 6]. Additionally, comparing the GGTs of these fields over 2008–2018 clearly showed that microbiome research is growing the fastest (two-tailed t-test; p = 0.007).

The analyses of these studies indicate that the GGT is a versatile measure to analyse publication trends that eliminates the bias effect of total publication growth, enables statistical trend correlations and growth rate comparisons for a specific field between periods or between different fields over the same period.

### Applying the GGT to analyse trends of well-known fields

**Fig 4:**
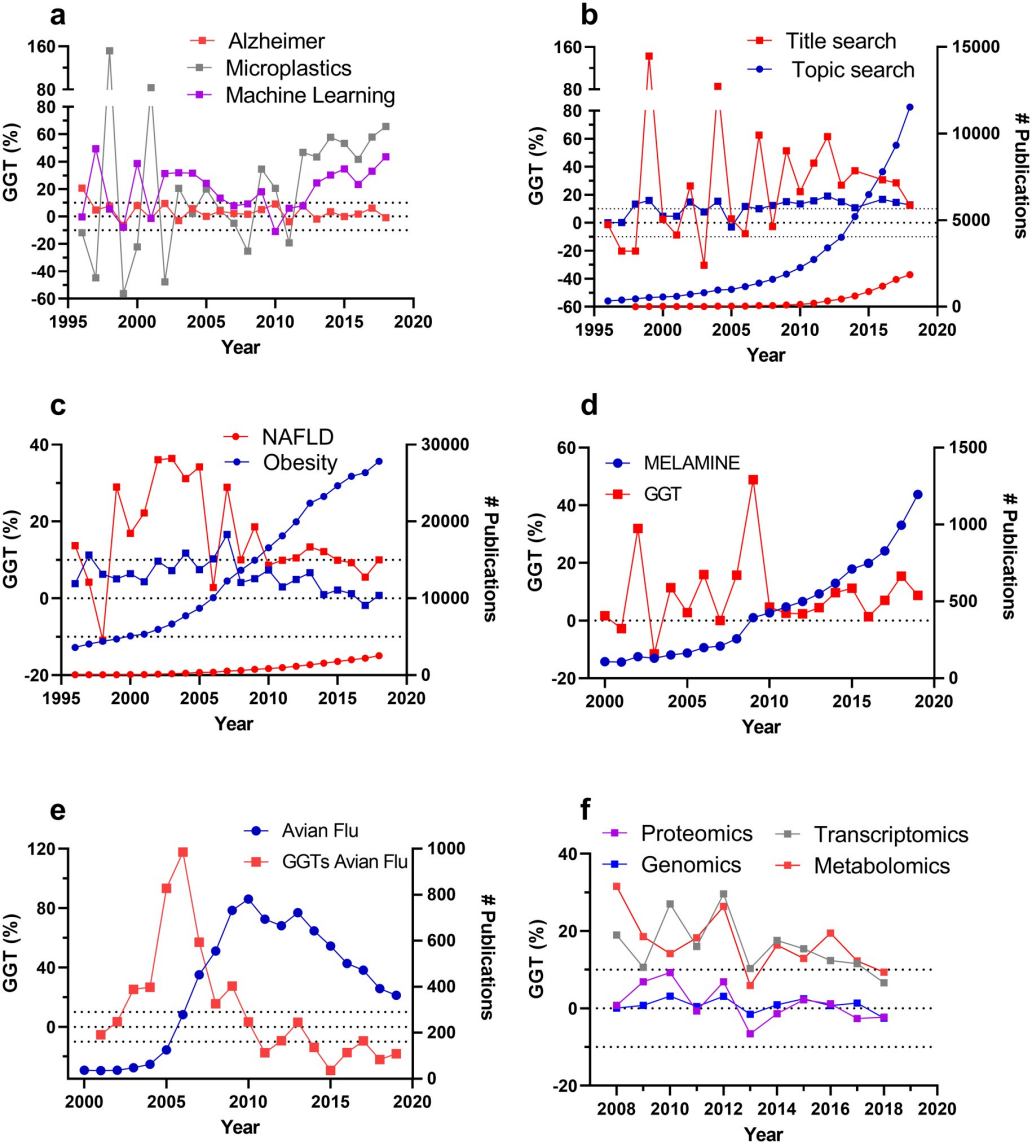
Trend analyses of publication trends on various life science fields. a) GGTs for the fields Alzheimer’s, microplastics and machine learning. b) GGTs (squares, left y-axes) and publication trends (circles, right y-axes) for the field microbiome using either a title search (red) or a field search (blue). c) GGTs (squares, left y-axes) and publication trends (circles, right y-axes) for the fields NAFLD and obesity. d) GGTs (red squares; left y-axes) and publication trends (blue circles; left y-axes) for melamine research. e) GGTs (red squares; left y-axes) and publication trends (blue circles; left y-axes) for the field avian flu (H5N1). f) GGTs for various omics techniques. All field searches were conducted in WoS excluding the ESCI index. Search strings and raw data used are given in the S2 File.

GGTs were calculated for publication trends obtained for 37 well-known fields (S2 File, Table 1) to investigate the potential of the metric in more detail. Fig 4 shows total publication trends and/or GGTs for the following fields: Alzheimer’s, microplastics, machine learning, microbiome, nonalcoholic fatty liver disease (NAFLD), obesity, melamine, avian flu, proteomics, transcriptomics, genomics and metabolomics. The first noteworthy aspect is that relatively young fields often have volatile GGTs with peaks that can be above 100%. This is likely due to the low number of publications coming out in the earlier years of these fields of study, making growth rates vulnerable to erratic change. Analysing publication trends in the early stages of a field’s development (below 50 papers yearly) will inevitably see large random fluctuations due to the larger proportional effect of random changes and if an inherent issue in publication trend analyses in emerging fields. However, this does not affect robustness when a slightly more developed stage is reached.

A good example is microplastics (Fig 4a) where an erratic GGT trend is observed over 2005–2011 (GGT = 4% ± 8%). In 2012 yearly publication output reached 41 publications and the GGTs stabilized (average GGT (2012–2018) = 51% ± 4%). Conversely, GGTs in well-established fields (such as Alzheimer’s Disease) fluctuate very little. Erratic behavior is also observed in the early years of other fields such as machine learning between 1996 and 2001, and other fields with few papers (<50) published per year as shown in S2 File (e.g. cell-based meat, edible insects or invasion biology). Thus, GGT values close to or above 100% seem to indicate that the field is relatively new. Additionally, it appears that GGT values above 100% indicate unsustainable growth. Therefore, as guiding principles, it is advised to interpret GGT values as reliable inflation corrected growth indicators only if:

GGTs are consistent (less than 50% year-to-year variation) and smaller than 100%

GGTs are calculated on a field with a yearly publication output of at least 50 publications per year

This said, if a field features GGTs around 100% or more with more than 50% year-to-year variation the GGT is still informative but more as a qualitative indication that the field is very young/undeveloped. Likewise, consistently growing fields with a <50 yearly publication output, are theoretically possible. GGT trends can also be classified into different sections.

We propose the following classification for consistent GGT values:

- Consistent decrease (GGTs < -10%).
- No consistent growth (-5%<GGTs<5%).
- Consistent growth (5%<GGT<15%).
- Consistent strong growth (15%<GGT<40%).
- Consistent intense growth 40%<GGT<80%.
- Unsustainable growth GGT>80%.

According to this classification system, the publication trends between 2013 and 2018 on Alzheimer’s disease can be classified as showing no consistent growth, while the fields microplastics and machine learning show consistently high growth during the same period (Fig 4a). Erratic GGT behavior may also indicate issues with the keyword string due to the sensitivity of growth rates to large relative fluctuations that may go unnoticed in the scatterplots showing yearly publication output. For instance, the GGTs for the field microbiome are erratic up to approximately 2010 with values well above 100 until 2005 when the search term as proposed in [6] was used even though this fluctuation does not show in the yearly publication output (Fig 4b). In this case, the conducted search only sought in titles for the keywords in the search string. When the same search string was used as a field search (searching within the title, abstract and keywords), the GGTs stabilized (GGT = 11% ± 1%; r = 0.36; p = 0.10) showing consistent, inflation-corrected growth.

The GGT can also help to display other information that remained hidden in the unadjusted publication trend. For example, the unadjusted publication trend for research on nonalcoholic fatty liver disease (NAFLD), one of the great public health concerns of the 20^th^ century, has consistently increased in volume since the late 1990s and the trend appears to mirror the trend for obesity (a major risk factor for NAFLD [24]) (Fig 4c). In contrast, the GGTs show a consistently strong growth trend in NAFLD research from 1999 to 2005 (GGT = 29% ±3%) before stabilizing, with a GGT of ~0.10 year on year while the GGT for obesity research (7% ± 1%) was significantly lower (t-test; p<0.0001) over that period. This difference would be hard to determine by looking at the basic publication trends. Another example of improved analyses using the GGT can be the detection of a brief burst of research activity following an outbreak or contamination event. For instance, the deliberate melamine contamination in Chinese infant milk powder in September 2008 [25] corresponds with the largest peak in the GGT in 2009 (~49%) (Fig 4d). Another example is the outbreak of avian flu (H5N1), which caused horizontal and secondary human transmission in 2003–2004. This outbreak coincides with an increase in the publication trend and GGT (Fig 4e). However, the GGT clearly peaks at 2006 at ~120% while the unadjusted publication trend appears to continue to increase at a similar rate until 2009. Therefore, changes in research intensity are more easily discerned using the GGT.

GGTs can be used to compare growth trends between related fields with disparate yearly publication outputs. Omics technologies have gained considerable attention over the past two decades due to their massive potential in a variety of fields including medicine, food authenticity and ecosystem health. However, it is difficult to quantifiably compare their growth trends due to the large difference in age and publication output. Using GGTs, it becomes clear that the research intensities for genomics (GGT = 0.8% ± 0.4%) are stable (r = -0.12, p = 0.73) and have ceased to grow between 2008–2018 (Fig 4f). Inflation-corrected growth for proteomics behaved similarly (GGT = 1.2% ± 1.4%) and showed (non-significant) indications of decline (proteomics r = -0.53, p = 0.10). Metabolomics and transcriptomics GGTs also appear to be on the decline between 2008 and 2018, although correlations are just not significant (metabolomics r = -0.57, p = 0.07; transcriptomics r = -0.51, p = 0.11). The average GGTs of these four fields are significantly different (one-way ANOVA p<0.0001). Specifically, the GGTs for metabolomics (GGT = 16% ± 2%) and transcriptomics (GGT = 17% ± 2%) were significantly higher than the GGTs for proteomics and genomics (Tukey post hoc p<0.0001) but no significant difference in the GGTs was observed between proteomics and genomics or between transcriptomics and metabolomics in post-hoc comparisons.

### Alternative relative growth corrections

**Fig 5:**
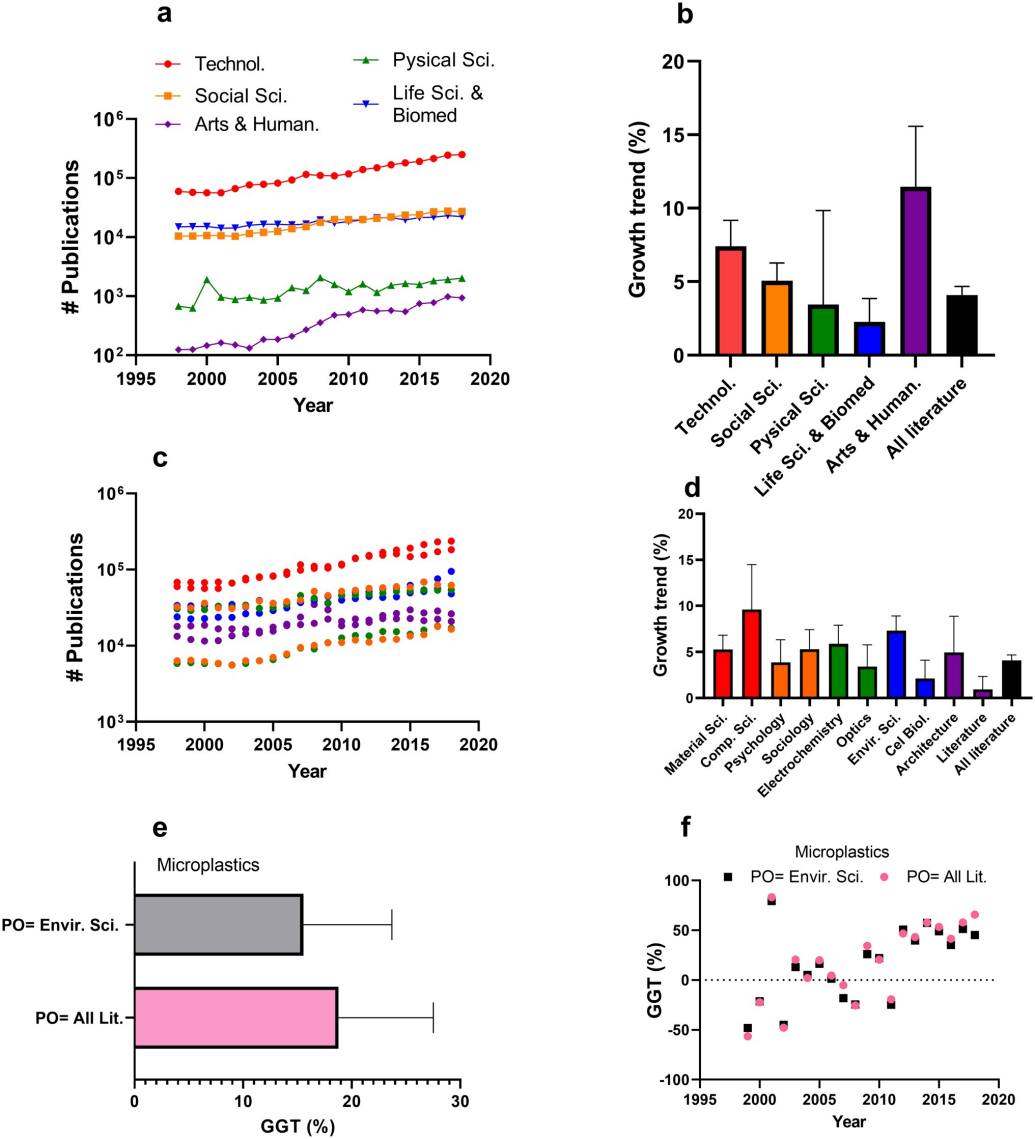
Alternative growth rate corrections. (a) shows total publication output for the five main research areas specified in WoS and (b) shows the growth rate calculated for those areas over the 2018–1998 period. (c) shows the total publications for WoS research categories (two categories per research area). (d) shows the growth rate calculated for those categories over the 2018–1998 period. Panel colours are matched to the research areas indicated in (a). The ESCI index was excluded for all analyses. (e) Average GGTs for the microplastics field using all literature or the WoS research category “environmental sciences” as the PO. (f) Annual GGTs for the field microplastics using all literature or the WoS research category “environmental sciences” as the PO.

The GGT is a relative measure of growth. Therefore, one needs to be careful when choosing the reference field, to make sure that GGTs are comparable. Moreover, one should clearly state this reference field to avoid confusion about what growth trends are being corrected for. The GGTs reported above describe growth in specific fields relative to the growth of the overall publication output on WoS. Such GGTs are a very useful and robust measure that enable to check if a specific field is growing faster as what would be expected from the average inflation observed across all fields in WoS. However, a more refined field specific inflation correction can also be considered to account for the difference in the frequency of publication between disciplines. Consider research fields a’ and b’ within disciplines A and B, where the publication frequencies of A and B are different. GGTs calculated for a’ and b’ would lead to unfair comparisons between the fields if publication frequency in A or B is significantly different from the overall publication frequency used to calculate PO. In such a scenario PO could be calculated individually for each discipline. The five major research areas (technology, social sciences, physical sciences, life sciences and biomedicine, and, arts & humanities) or the major categories used in WoS could be used to get discipline specific inflation corrections to overcome this issue. The publication and growth trends of the WoS research areas and WoS research categories are shown over the 2018–1998 period (Fig 5; S2 File). A One-way ANOVA was not significant for research areas (p = 0.38). Thus, making this distinction for PO calculation may not result in highly different results. That said, arts & humanities did have a slightly higher (non-significant) growth rate over this period than all literature (p = 0.085; two-tailed t-test) while the life sciences and biomedicine growth rate was similar to all literature (p = 0.29; two-tailed t-test). The absolute number of publications produced in these fields is very different, with arts & humanities producing less publications per year. This scale difference may result in different growth rates of these fields, as yearly variation in PO in arts & humanities will cause larger changes to the growth trend. Thus, research area specific PO calculation may be useful to obtain GGTs that are corrected for interdisciplinary differences in publication practices between fields and detail the growth of a field relative to the overall growth in that research area instead of to all literature. However, we do not expect this to make large differences to the overall analyses in this case since no statistical differences in the growth rates of individual research areas compared to the growth rate of all literature was observed. In some cases, growth rates of individual research categories may differ, and could be used for PO instead to obtain growth rates for a field relative to its respective research category. The one-way ANOVA between all WoS categories tested did not show a statistically significant effect of category (p = 0.55) (Fig 5d). Post-hoc analyses comparing the growth rates of all the WoS categories with the total publication growth rate did not find significant differences, with p>0.90 in all cases except for the comparison of all literature versus computer science (p = 0.58). Thus, it was considered here that PO calculation using all literature does not introduce large bias in the GGT calculations for the topics discussed (which fall in the research categories investigated). However, a t-test comparing the growth in the environmental sciences category with the growth of all literature showed near significantly different growth rates (p = 0.06; two-tailed t-test) (Fig 5c and 5d). Thus, when investigating the growth of a specific field in this category (e.g., microplastics), one could investigate if that field is growing relative to the growth in all literature and relative to the growth in the environmental sciences category using both a general “total publication” PO (correction a’) and a WoS category specific PO (correction b’). If GGT using correction a’ are significantly different from GGTs using correction b’ then this would indicate that the observed differences in growth trends between the fields are, at least partly, due to differences between the higher-level research categories, rather than being field-specific differences. Additionally, correlations using a’ and b’ can be calculated to further investigate the relative growth of a field. Such detailed analyses with variable PO use can help to identify confounding factors and obtain more precise comparisons of specific research fields. For microplastics (a field in the environmental science category) the average GGT relative to the environmental sciences research area was 15.6 ± 8.0% for the period between 2018 and 1998 whereas the average GGT relative to all literature was 18.8 ± 8.0% (Fig 5e). This difference is not significant (p = 0.79; two-tailed t-test). Moreover, correlation coefficients were similar (r = 0.63, p = 0.003, PO = all literature; r = 0.59, p = 0.007, PO = environmental sciences) (Fig 5f). Thus, in the case of microplastics, one can conclude that the field is growing significantly faster as the overall literature and the environmental research category. Overall, using a different PO can be useful to investigate the growth of a field relative to the growth in a specific discipline and to compare fields from disciplines that have discipline specific growth-rates that substantially vary from the overall growth rate of all literature. That said we did not detect any cases in our analyses where research categories or research areas differed sufficiently from the overall growth rate of all literature to cause large differences in GGTs, further showing the robustness of the GGT for trend analyses.

### Perspectives for inflation corrected growth rates

**Fig 6:**
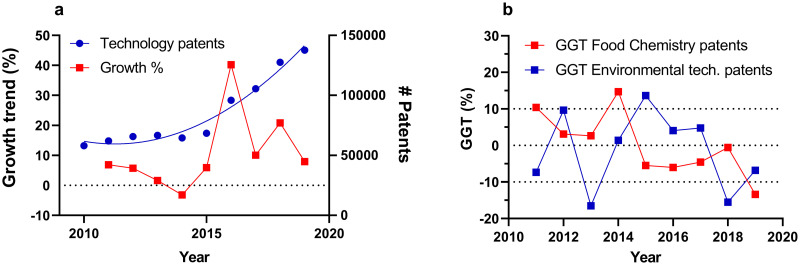
Patent trend analyses. a) Total yearly technology patent output (blue circles; right y-axes) and yearly technology patent output growth rate percentages (red squares, left axes). b) GGTs for food chemistry patents (red squares) and environmental technology patents (blue squares). Patent data was downloaded from the EPO database.

Other applications include correcting the growth in a specific research field for the growth observed in another reference field as total publication output. The principles behind the GGT would remain identical but changing the reference field may help to compare field-specific growth with the growth of the related overarching field. This enables more in-depth investigations. For example, it would enable visualizing the increased application of machine learning tools to Alzheimer’s-related fields relative to the growing use of machine learning methods in the overall scientific literature. Inflation-corrected growth trends can also be calculated on changes in patent growth for various European Patent Office (EPO) categories. Total technology patents reported (2010–2019) in the EPO database have increased, and a quadratic polynomial trendline can be fitted to the data (R^2^ = 0.97; Fig 6a). Moreover, growth rates have been relatively stable except for a clear spike in the year 2016, possibly indicating a change in the database indexing system similar to what was observed for WoS. Interestingly, the GGTs visualised in Fig 6b for the EPO food chemistry and environmental technology patents show that inflation-corrected food chemistry patent registration has significantly decreased (r = -0.75; p = 0.026). Patent submissions for environmental technology, on the other hand, have been on par with the total growth in technology patents (r = -0.02, p = 0.98).

## Conclusion

The GGT is a versatile measure that quantifies field-specific growth rates after removing the bias caused by the overall growth of the general publication output. The measure enables statistical comparison between separate research fields of similar or different dimensions. That said, caution remains key when making comparisons between fields due to field differences regarding field specific co-authorship practises, publication output type (e.g. short conference papers or lengthy journal articles) and the field-specific differences in the need for time and resource consuming laboratory experiments. The GGT does not correct for these differences which should be considered when comparing field-specific growth rates from dissimilar research fields. It is possible to perform statistical analyses to discern trends and detect brief changes in the growth tendency of a research field caused by a temporary increase in research intensity. The reference field of GGTs can be used to calculate growth rates relative to the growth in specific research fields or can be calculated on field-specific patent publication to inform on relative growth rates for patent production as well. The measure can be most informative if accompanied by unadjusted publication trends thus enabling statistical analyses of inflation corrected growth and appreciation of the overall size of the studied research field. Such analyses will provide a more detailed, quantitative and sound temporal analyses of the actual changes in field-specific research intensity. Better-quality trend analyses may considerably improve the identification of key knowledge gaps and highlight the commercial potential of emerging technology. This will ultimately result in more efficient and evidence-driven decision-making regarding research effort investment and funding allocation, as true growth is better visualized and quantified.

## Supporting information

S1 File The data and calculations used to reanalyse trends reported in reviews. (XLSX)

S2 File The data and calculations used for trend analyses of an additional 37 fields and for determining the effect of changing the article types or PO criteria for PO calculations. (XLSX)

S3 File An excel template to calculate novel GGT values for any given field. (XLSX)

S4 File The R code that can be used to calculate GGTs. (R)

S5 File Operation instructions to use the R script. (PDF)
