# Supplementary material for: The General Growth Tendency: A tool to improve publication trend reporting by removing record inflation bias and enabling quantitative trend analysis
Source: PLoS One. 2022 May 20;17(5):e0268433. doi: 10.1371/journal.pone.0268433 (PMC9122180; doi:10.1371/journal.pone.0268433)
Supplement: S5 File — (PDF) [file pone.0268433.s005.pdf]

GGT calculation

This is an R markdown document providing an R function to calculate the GGT function, and an example of how to use it.

$$GGT = 100 * (\frac{PS_y - PS_{y-1}}{PS_{y-1}} - \frac{PO_y - PO_{y-1}}{PO_{y-1}})$$

GGT calculation function

Select the entire function and run it to load it in the workspace.

```
calculate.GGT = function(year,PS,PO,period) {

	N = length(PS)

	growth.data = matrix(data = 0, nrow = N, ncol = 2)
	for(i in 2:N) {
		growth.data[i,1] = (PS[i]-PS[i-1])/PS[i-1]
		growth.data[i,2] = (PO[i]-PO[i-1])/PO[i-1]
	}

	GGT = 100*(growth.data[,1]-growth.data[,2])

	output1 = data.frame(year,GGT)

	output = output1
	if(period>1){
		for(i in period:N) {
			output[i,2] = mean(output1[(i-(period-1)):i,2])
		}
	}

	output = output[-(1:period),]

	output[,1] = paste0(year[1:(N-period)],"-",year[(1+period):N])
	return(output)
}
```

Working example

```r
# load hypothetical data
year = c(2000, 2001, 2002, 2003)
subfield = c(1488, 1623, 1620, 2161)
field = c(831188, 831250, 850273, 887909)

# these data can be loaded as vectors like this, or they can be imported from csv, txt or xlsx files
# using functions read.csv(), read.table(), or read_xls() (requires package readxl)

# calculate annual GGT
annual.GGT = calculate.GGT(year,PS = subfield,PO = field,period=1)
annual.GGT
```

```
##        year       GGT
## 2 2000-2001  9.065121
## 3 2001-2002 -2.473324
## 4 2002-2003 28.968719
```

```r
# calculate biennial GGT
biennial.GGT = calculate.GGT(year,PS = subfield,PO = field,period=2)
biennial.GGT
```

```
##        year       GGT
## 3 2000-2002  3.295899
## 4 2001-2003 13.247697
```

```r
# to export these data, a function like write.csv() or write.table() can be used
# e.g. write.csv(annual.GGT,"annualGGT.csv")
```
